# Acute Functional Colonic Dysmotility Mimicking Mechanical Bowel Obstruction Following Self-Limited Diarrheal Illness: A Case Report

**DOI:** 10.7759/cureus.103684

**Published:** 2026-02-15

**Authors:** Saad B Sadiq

**Affiliations:** 1 General Practice, Lifeline Hospital Salalah, Salalah, OMN

**Keywords:** constipation, diarrhea, fecal impaction, functional obstruction, intestinal obstruction, laxative, mechanical obstruction, post-infectious complications

## Abstract

Acute constipation following diarrheal illness is commonly encountered and is often benign. However, when absolute constipation persists despite adequate hydration, preserved mobility, and failure of multiple laxative classes, it can closely mimic mechanical bowel obstruction and create significant diagnostic uncertainty. We report a case of a 56-year-old female with a previously normal daily bowel habit who developed acute obstipation following a self-limited diarrheal episode, unresponsive to bulk-forming, osmotic, and stimulant laxatives. Despite adequate oral intake and activity, symptoms persisted for five days with reduced flatus and progressive abdominal distension. Escalation to contrast-enhanced computed tomography demonstrated colonic fecal loading without evidence of mechanical obstruction. The patient was successfully managed conservatively with rectal enemas and supportive care. This case highlights that functional colonic dysmotility can present with absolute constipation refractory to standard measures and may convincingly mimic surgical pathology, underscoring the importance of structured diagnostic escalation.

## Introduction

Acute constipation is a frequent presentation in primary care and emergency settings and is typically functional and self-limiting [1]. Standard management includes hydration, mobility, and the use of bulk-forming or osmotic laxatives, with most cases responding promptly [1,2]. However, when constipation presents as obstipation with reduced flatus, abdominal distension, and failure of multiple conservative therapies, concern for mechanical bowel obstruction is appropriately raised [3].

Functional colonic dysmotility refers to impaired colonic propulsion in the absence of a fixed anatomical obstruction. Clinically, this can mimic mechanical large bowel obstruction, which is characterized by a structural transition point or obstructing lesion visible on imaging [3]. It must also be distinguished from post-infectious irritable bowel syndrome, which represents a chronic functional disorder rather than an acute obstructive phenotype [4], and from acute colonic pseudo-obstruction (Ogilvie syndrome), which typically occurs in hospitalized or systemically unwell patients with marked colonic dilatation and identifiable precipitating factors [5].

Post-infectious disturbances of colonic motility are recognized contributors to transient bowel dysfunction and may reflect inflammatory-mediated neuromuscular alterations following enteric infection [4,6]. Acute colonic pseudo-obstruction has also been described in association with severe systemic illness and impaired autonomic regulation [7]. In particular, an abrupt transition from self-limited diarrhea to absolute constipation in a patient with previously normal bowel habits can generate significant diagnostic uncertainty. This case illustrates how acute functional colonic dysmotility can present with features suggestive of mechanical obstruction, prompting escalation to advanced imaging and underscoring the importance of confidently excluding structural pathology before pursuing invasive intervention.

## Case presentation

A 56-year-old female presented with progressive abdominal distension, constipation, and inability to pass flatus for five days. She reported a previously normal daily bowel habit. Her symptoms were preceded by a short episode of acute diarrhea lasting approximately 24-48 hours, which resolved spontaneously without antimicrobial or antimotility therapy. The Bristol Stool Chart type was not formally recorded. She reported increased stool frequency during the diarrheal episode. Constipation began immediately after the resolution of the diarrheal illness. She denied nausea, vomiting, fever, gastrointestinal bleeding, weight loss, anorexia, or prior similar episodes. There was no history of abdominal surgery, chronic gastrointestinal disease, or use of opioids, anticholinergic agents, antidepressants, calcium channel blockers, or other medications known to impair bowel motility.

Despite preserved mobility and adequate oral hydration, early use of multiple conservative measures failed to produce a bowel movement. She self-administered macrogol (one sachet daily for two days), lactulose (15 mL twice daily for three days), and a stimulant laxative (bisacodyl 10 mg at night for two nights).

On examination, the patient appeared uncomfortable but was hemodynamically stable and afebrile. Vital signs were as follows: blood pressure at 126/70 mmHg, pulse at 74 bpm, temperature was normal (36.6°C), and SpO₂ was 100% on room air. The abdomen was mildly and uniformly distended with hyper-tympanitic percussion. Distension progressed gradually over five days as reported by the patient; objective serial abdominal girth measurements were not recorded. She reported mild, continuous, non-colicky abdominal discomfort localized to the left iliac fossa without radiation. Tenderness was mild and localized, without guarding, rigidity, or rebound tenderness. Bowel sounds were present and hyperactive. Digital rectal examination did not reveal fecal impaction. Flatus had progressively reduced to complete absence.

Laboratory investigations were largely unremarkable and are summarized in Table 1. There was no evidence of electrolyte imbalance, renal or hepatic dysfunction, endocrine abnormality, or systemic infection. Serum calcium was not measured; however, there were no clinical features suggestive of hypercalcemia. C-reactive protein was mildly elevated (5.85 mg/L) in the absence of leukocytosis or systemic inflammatory features.

**Table 1:** Summary of laboratory investigations.

Test (unit)	Observed value	Reference range
Hemoglobin (g/dL)	13.5	11–15
Total white blood cell count (cells/mm³)	4,710	4,000–11,000
Platelets (×10³/mm³)	230	150–450
C-reactive protein (mg/L)	5.85	<5
Blood urea (mg/dL)	21.3	15–45
Serum creatinine (mg/dL)	0.87	0.6–1.1
Sodium (mmol/L)	145.7	135–145
Potassium (mmol/L)	4.1	3.5–5.1
Aspartate aminotransferase (U/L)	15.9	0–31
Alanine aminotransferase (U/L)	14.4	0–32
Alkaline phosphatase (U/L)	94.2	40–129
Thyroid-stimulating hormone (μIU/mL)	0.80	0.27–4.2

**Figure 1:**
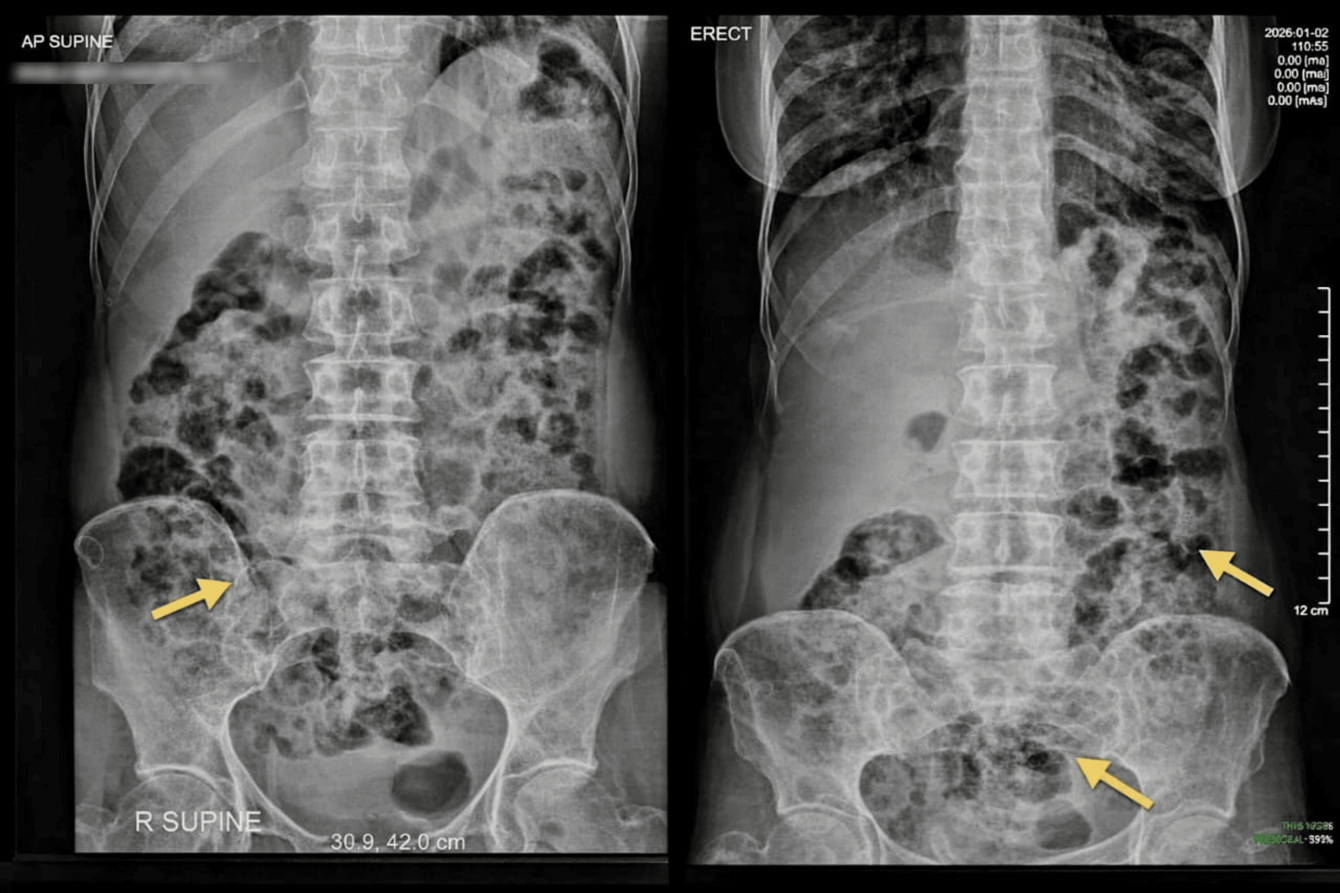
Abdominal radiographs demonstrating colonic fecal loading. Supine and erect abdominal radiographs show diffuse colonic gaseous distension with prominent fecal loading (arrows). There are no air-fluid levels, free sub-diaphragmatic air, or radiographic features of a transition point.

Plain abdominal radiographs (supine and erect views) demonstrated diffuse colonic gaseous distension with fecal loading, without air-fluid levels or free sub-diaphragmatic air (Figure 1). Abdominal ultrasonography showed excessive bowel gas with probe tenderness but no solid organ pathology or sonographic evidence of obstruction.

Given the persistence of absolute constipation and failure of multiple conservative therapies, a general surgical opinion was sought, and a standard contrast-enhanced CT of the abdomen and pelvis (intravenous contrast; no oral contrast) was performed. CT demonstrated diffuse colonic fecal loading and gaseous distension without evidence of a transition point, obstructing lesion, volvulus, bowel wall thickening, ischemia, or perforation.

Following exclusion of mechanical obstruction on contrast-enhanced CT, the patient was managed conservatively with rectal sodium phosphate enemas, hydration, and supportive care, alongside close clinical observation. Rectal therapy was prioritized following failure of oral laxatives to directly evacuate distal fecal loading. Bowel function returned gradually, with progressive passage of stool and flatus and resolution of abdominal distension. No pharmacologic prokinetic therapy or invasive intervention was required.

## Discussion

This case illustrates the diagnostic challenge posed by acute functional constipation presenting with features classically associated with mechanical bowel obstruction. When obstipation, absence of flatus, and progressive abdominal distension coexist, concern for structural obstruction is appropriately heightened [3,5]. In contrast to typical functional constipation, this patient developed abrupt absolute constipation following a brief diarrheal illness, despite preserved mobility, adequate hydration, and failure of multiple laxative classes. This constellation reasonably justified escalation to advanced imaging to exclude obstructive pathology [1-3].

Functional colonic dysmotility refers to impaired colonic propulsion in the absence of a fixed anatomical obstruction. Acute disturbances in colonic motor activity have been described following enteric infections and may reflect transient inflammatory or neurogenic alterations in enteric signaling [4,6]. In this case, the immediate transition from self-limited diarrhea to obstipation supports a transient functional disturbance rather than progressive chronic constipation. However, the precise pathophysiological mechanism cannot be definitively established in a single case, and the diagnosis remains clinical following exclusion of mechanical causes.

The mildly elevated C-reactive protein observed was nonspecific and most consistent with recent physiological or post-inflammatory stress, particularly given the absence of leukocytosis, systemic features, or radiologic inflammatory changes. Similarly, medication-related hypomotility was excluded based on history, and no biochemical or structural abnormalities were identified to suggest alternative causes of obstruction.

Importantly, this case underscores the value of a structured diagnostic approach in patients with acute severe constipation accompanied by red-flag features. Contrast-enhanced computed tomography plays a central role in confidently excluding mechanical obstruction, ischemia, and other surgical pathology, thereby allowing safe conservative management [3,5]. Recognition that functional obstruction can mimic surgical disease helps prevent both delayed diagnosis of true obstruction and unnecessary invasive intervention in appropriately selected patients.

Learning point

Acute functional colonic dysmotility can closely mimic mechanical large bowel obstruction. In patients presenting with acute obstipation and abdominal distension, structural pathology must be confidently excluded before a functional diagnosis is made.

## Conclusions

Acute functional colonic dysmotility may present with absolute constipation refractory to standard conservative measures and convincingly mimic mechanical bowel obstruction. In patients with obstipation, abdominal distension, and reduced flatus, structural pathology must be confidently excluded before a functional diagnosis is established. Timely use of contrast-enhanced computed tomography facilitates safe differentiation between functional and mechanical causes and supports appropriate conservative management. This case underscores the importance of structured clinical reasoning when evaluating severe constipation with red-flag features.
